# Supplementary material for: J-Shaped Relationship of Serum Uric Acid with Unfavorable Short-Term Outcomes among Patients with Acute Ischemic Stroke
Source: Biomedicines. 2022 Sep 4;10(9):2185. doi: 10.3390/biomedicines10092185 (PMC9496357; doi:10.3390/biomedicines10092185)
Supplement: Supplementary file 1 [file biomedicines-10-02185-s001.zip › biomedicines-1877652-supplementary.pdf]

Table S1. Comparison of measured variables with different quartiles of uric acid derived from each group among all patients, male and female patients

	Quartile 1	Quartile 2	Quartile 3	Quartile 4	*p*-Value
All patients (*n* = 3370)					
Uric acid (mg/dL)	<4.1	4.1-5.1	5.2-6.2	>6.2	
Number of patients	889	847	811	823	
Age (years)	71.0±13.6	70.3±13.5	70.4±13.3	70.8±14.1	0.619
Female gender	510 (57)	386 (46)	280 (35)	269 (33)	<0.001
NIHSS score on admission	7.5±7.2	6.4±6.9	6.3±6.6	7.5±7.9	<0.001
mRS score >2 at discharge	510 (57)	386 (46)	280 (45)	403 (49)	<0.001
Death at discharge	40 (4.5)	26 (3.1)	19 (2.3)	48 (5.8)	0.001
Male patients (*n* = 1925)					
Uric acid (mg/dL)	<4.4	4.4-5.4	5.5-6.5	>6.5	
Number of patients	453	585	435	452	
Age (years)	69.1±13.5	68.1±13.1	67.6±13.6	67.6±13.9	0.293
NIHSS score on admission	6.0±6.1	5.8±6.1	5.6±6.2	6.9±7.5	0.009
mRS score >2 at discharge	219 (48)	247 (42)	157 (36)	195 (431)	0.003
Death at discharge	25 (5.5)	15 (2.6)	7 (1.6)	21 (4.6)	0.003
Female patients (*n* = 1445)					
Uric acid (mg/dL)	<3.8	3.8-4.6	4.7-5.9	>5.9	
Number of patients	356	347	399	343	
Age (years)	72.3±13.5	73.5±13.1	73.3±12.9	77.3±11.9	<0.001
NIHSS score on admission	8.8±8.0	7.8±7.6	7.3±736	8.9±8.4	0.004
mRS score >2 at discharge	218 (61)	190 (55)	214 (54)	218 (64)	0.015
Death at discharge	9 (2.5)	16 (4.6)	15 (3.8)	25 (7.3)	0.020

data are expressed as mean ± standard deviation or n (%); mRS, modified Rankin Scale; NIHSS, National Institutes of Health Stroke Scale

Table S2. Multivariable analysis of factors influencing unfavorable outcomes (modified Rankin Scale score > 2) in 3370 patients with acute ischemic stroke including quartile distribution of uric acid derived from each group.

Characteristics	All patients (*n* = 3370)		Male patients (*n* = 1925)		Female patients (*n* = 1445)	
	OR (95% CI)	Model I *p*-Value	OR (95% CI)	Model I *p*-Value	OR (95% CI)	Model I *p*-Value
Age	1.051 (1.042-1.061)	<0.001	1.045 (1.033-1.057)	<0.001	1.054 (1.040-1.069)	<0.001
Admission NIHSS score	1.422 (1.377-1.467)	<0.001	1.434 (1.377-1.493)	<0.001	1.409 (1.342-1.479)	<0.001
Female gender	1.200 (0.955-1.508)	0.110	-	-	-	-
Hemoglobin	0.952(0.899-1.008)	0.147	0.923 (0.859-0.993)	0.031	0.991 (0.997-1.001)	0.850
Platelet	0.999 (0.998-1.001)	0.424	1.000 (0.998-1.002)	0.777	0.999 (0.997-1.001)	0.445
White blood cells	1.058 (1.016-1.102)	0.005	-	-	1.130 (1.059-1.206)	<0.001
Glucose	1.002 (1.001-1.004)	0.012	-	-	1.003 (1.000-1.005)	0.043
Creatinine	0.978 (0.862-1.110)	0.929	0.891 (0.763-1.040)	0.143	1.072 (0.926-1.241)	0.352
Cholesterol	1.003 (1.001-1.006)	0.014	1.004 (1.001-1.008)	0.018	1.003 (0.999-1.007)	0.132
Triglyceride	1.000 (0.999-1.001)	0.509	1.001 (0.999-1.002)	0.361	0.999 (0.997-1.002)	0.647
Hypertension	1.024 (0.825-1.270)	0.657	-	-	1.131 (0.799-1.601)	0.487
Diabetes mellitus	1.147 (0.904-1.455)	0.309	1.397 (1.081-1.805)	0.011	1.026 (0.697-1.511)	0.896
Dyslipidemia	0.837 (0.653-1.072)	0.218	0.949 (0.689-1.306)	0.747	0.778 (0.531-1.139)	0.196
Heart disease	0.888 (0.711-1.110)	0.428	0.985(0.744-1.304)	0.916	0.848 (0.595-1.207)	0.359
Prior stroke	1.574 (1.254-1.977)	<0.001	1.695 (1.285-2.235)	<0.001	1.506 (1.033-2.196)	0.033
Current smoker	1.023 (0.787-1.328)	0.838	1.054 (0.813-1.366)	0.691	0.625 (0.290-1.344)	0.213
Alcohol consumption	0.926 (0.618-1.388)	0.688	-	-	-	-
History of cancer	1.655 (1.139-2.405)	0.011	-	-	1.920 (1.102-3.346)	0.021
Uremia	1.492 (0.557-3.994)	0.670	2.659 (0.767-9.219)	0.123	-	-
Quartile 1 of uric acid[a]	1.359 (1.068-1.730)	0.013	1.413 (1.053-1.895)	0.021	1.217 (0.832-1.781)	0.312
Quartile 4 of uric acid[a]	0.972 (0.766-1.233)	0.815	0.980 (0.718-1.339)	0.901	1.001 (0.684-1.466)	0.994

Model I: including uric acid quartiles 1 to 4, range of Quartiles 1 and 4 for all patients: <4.1 mg/dL and >6.2 mg/dL, for male patients: <4.4 mg/dL and >6.5 mg/dL, for female patients: <3.8 mg/dL and >5.9 mg/dL, respectively; [a]Using Quartiles 2 and 3 of uric acid as reference percentiles; CI, confidence interval; NIHSS, National Institutes of Health Stroke Scale; OR, odds ratio

Table S3. Multivariable analysis of factors influencing death in 3370 patients with acute ischemic stroke including quartile distribution of uric acid derived from each group.

Characteristics	All patients (n = 3370)		Male patients (n = 1925)		Female patients (n = 1445)	
	OR (95% CI)	Model I p-Value	OR (95% CI)	Model I p-Value	OR (95% CI)	Model I p-Value
Age	1.019 (1.001-1.036)	0.025	1.023 (1.000-1.046)	0.047	1.025 (0.996-1.054)	0.091
Admission NIHSS score	1.134 (1.111-1.157)	<0.001	1.165 (1.130-1.201)	<0.001	1.127 (1.093-1.162)	<0.001
Hemoglobin	0.952 (0.862-1.053)	0.360	0.887 (0.772-1.020)	0.092	-	-
Platelet	-	-	1.001 (0.998-1.004)	0.532	-	-
White blood cells	1.110 (1.049-1.176)	<0.001	1.136 (1.040-1.241)	0.005	1.088 (1.005-1.178)	0.038
Glucose	1.002 (1.000-1.004)	0.078	-	-	1.001 (0.998-1.004)	0.459
Creatinine	1.064 (0.883-1.283)	0.472	-	-	1.208 (1.028-1.420)	0.022
Triglyceride	-	-	1.001 (0.997-1.004)	0.751	-	-
Diabetes mellitus	-	-	-	-	1.525 (0.821-2.832)	0.182
Heart disease	1.844 (1.205-2.820)	0.004	1.290 (0.700-2.377)	0.415	2.356 (1.234-4.499)	0.009
History of cancer	2.100 (1.114-3.958)	0.033	-	-	2.120 (0.923-4.872)	0.077
Uremia	1.410 (0.376-5.295)	0.669	-	-	-	-
Quartile 1 of uric acid[a]	1.474 (0.895-2.427)	0.127	3.198 (1.595-6.411)	0.001	0.490 (0.217-1.109)	0.087
Quartile 4 of uric acid[a]	1.498 (0.942-2.381)	0.088	1.610 (0.776-3.337)	0.201	1.128 (0.604-2.110)	0.705

Model I: including uric acid quartiles 1 to 4; range of Quartiles 1 and 4 for all patients: <4.1 mg/dL and >6.2 mg/dL, for male patients: <4.4 mg/dL and >6.5 mg/dL, for female patients: <3.8 mg/dL and >5.9 mg/dL, respectively; [a]Using Quartiles 2 and 3 of uric acid as reference percentiles; CI, confidence interval; NIHSS, National Institutes of Health Stroke Scale; OR, odds ratio
